# Statistical considerations in long-term efficacy evaluation of anti-cancer therapies

**DOI:** 10.3389/fphar.2023.1265953

**Published:** 2023-10-03

**Authors:** Ruobing Li, Jingyi Zhang, Jingzhao Wang, Jun Wang

**Affiliations:** ^1^ Office of Biostatistics and Clinical Pharmacology, Center for Drug Evaluation, National Medical Products Administration, Beijing, China; ^2^ Research Center of Biostatistics and Computational Pharmacy, China Pharmaceutical University, Nanjing, China

**Keywords:** anti-cancer therapy, long-term efficacy evaluation, estimand, patient-reported outcomes, non-proportional hazard assumption, treatment switching

## Abstract

Anti-cancer therapy has been a significant focus of research. Developing and marketing various types and mechanisms of anti-cancer therapies benefit a variety of patients significantly. The long-term benefit to patients in evaluating the risk-benefit ratio of anti-cancer therapy has become a significant concern. This paper discusses the evaluation of long-term efficacy within the estimand framework and summarizes the various strategies for addressing potential intercurrent events. Non-proportional hazards of survival data may arise with novel anti-cancer therapies, leading to potential bias in conventional evaluation methods. This paper reviews statistical methods for addressing this issue, including novel endpoints, hypothesis testing, and efficacy estimation methods. We also discuss the influences of treatment switching. Although advanced methods have been developed to address the non-proportional hazard, they still have limitations that require continued collaborative efforts to resolve issues.

## 1 Introduction

As one of the most severe threats to human health, cancer has long captured the attention of industry, academia, and regulatory authorities, driving continuous efforts to develop effective anti-cancer treatment therapies. Notably, from 2010 to 2019, 25% of drugs approved by the FDA were anti-cancer drugs (Brown and Wobst, 2021). The past decade has been coined the “Golden Age of Oncology” (Landau, 2019), witnessing the rapid emergence and approval of diverse anti-cancer treatments, including immunotherapy, targeted therapies, and cell therapies. From 2000 to 2022, 206 anti-cancer treatments received approval for 573 indications, including 50 cytotoxic drugs, 277 targeted drugs, and 246 targeted biologics (Scott et al., 2023). Among these, targeted drugs and biologics have become the leading modalities in modern anti-cancer medications.

Recent research reveals that newly approved anti-cancer drugs are crucial in contemporary real-world cancer treatment. From 1 May 2016, to 31 May 2021, 28 drugs were approved as first-line anti-cancer therapies, 32 as first-line alternative therapies, and 86 as second-line therapies (Benjamin et al., 2022). Furthermore, developing and using various anti-cancer therapies have significantly improved tumor remission and long-term patient benefits (Hoos, 2016). An extensive analysis of cancer patient survival in China revealed a remarkable increase in survival rates from 2003 to 2015, indicating advancements in cancer care standards. Notably, the age-standardized 5-year relative survival for all cancer types combined demonstrated substantial growth from 30.9% to 40.5% from 2003–2005 to 2012–2015 (Zeng et al., 2018).

In evaluating the efficacy of anti-cancer drugs, accelerated approvals often rely on short-term outcomes, such as objective response rate (ORR) and disease control rate. Whereas full approvals usually need positive results in long-term outcomes, such as progression-free survival (PFS) and overall survival (OS). While a drug may receive accelerated approval for market entry based on promising outcomes of short-term endpoints, conducting rigorous randomized controlled studies to demonstrate significant long-term benefits remains essential to obtain full approval. The failure to demonstrate long-term benefits can lead to the withdrawal of the drug from the market (Scott et al., 2023). Therefore, reliable long-term benefit is crucial in determining the efficacy of anti-cancer drugs. However, due to the diverse mechanisms of action of anti-cancer drugs and their complex interactions, the conventional efficacy evaluation methods may not be entirely applicable. For example, immunotherapy exhibits unique characteristics at both cellular and systemic levels compared to other anti-cancer drug treatments, leading to intricate phenomena such as delayed effects (Finn, 2012; Borcoman et al., 2019). Therefore, conducting comprehensive and flexible evaluations of the appropriateness of conventional statistical methods when assessing the efficacy of different anti-cancer drugs is essential.

This paper will discuss practical considerations in assessing the long-term efficacy of anti-cancer therapies, while details of conventional methods are beyond the scope of this paper. The paper is structured as follows: Section 2 reviews the significance of estimand and its application in long-term efficacy evaluation. Section 3 investigates the selection of endpoints for assessing long-term efficacy, including comparing short-term and long-term endpoints, novel long-term endpoints, and patient-reported outcomes (PROs). Section 4 discusses statistical considerations for evaluating long-term efficacy, including non-proportional hazards (NPH) assumptions, hypothesis testing, efficacy estimation, and treatment switching. We conclude with a brief discussion in Section 5.

## 2 Estimand

### 2.1 Estimand: the attributes and significance

The primary objective of clinical drug development is to establish the efficacy and safety of drugs through a series of clinical trials. The information confirmed through these trials is subsequently incorporated into drug labels to instruct physicians and patients. However, different stakeholders (sponsors, physicians, patients, regulatory authorities) may hold different interpretations of a drug’s treatment effect. This disparity can impact trial design, implementation, evaluation, and interpretation of clinical efficacy outcomes.

The International Council for Harmonisation of Technical Requirements for Pharmaceuticals for Human Use (ICH) drafted E9(R1): “Estimating Objectives and Sensitivity Analysis in Clinical Trials” in October 2014. This addendum guides precise definitions and accurate descriptions of the treatment effects within trial protocols. E9(R1) proposes a structured framework to help clarify the objective, design, implementation, analysis, and interpretation of trials. This framework facilitates efficient dialogue among various stakeholders involved in clinical trials and enhances clarity regarding treatment effects in clinical trials. To align domestic drug registration standards with international practices, many countries worldwide, including the United States and European Union member states, incorporate the principles outlined in E9(R1) into their regulatory frameworks for clinical trials. For example, China’s National Medical Products Administration (NMPA) mandated the implementation of ICH E9(R1) in drug clinical research, effective from 25 January 2022. To foster industry comprehension and implementation of “estimand” and “sensitivity analysis,” the DIA China Statistics Community collaborated with domestic industry experts to develop a bluebook. This document includes detailed explanations of these concepts and case studies to promote comprehension and application of E9(R1) in China.

The principle of E9(R1) is constructing a framework that harmonizes the alignment between trial objectives and implementation. This framework fosters thoughtful considerations and effective communication among diverse stakeholders. The framework consists of three steps. The first step involves clearly defining the trial objective based on the specific clinical question of interest. Before initiating the clinical trial, it is crucial to comprehend the indications and characteristics of the drug thoroughly, as well as evaluate the potential intercurrent events (ICEs) that may impact efficacy assessment. This initial step forms the foundation for setting the estimand, constituting the subsequent steps. The second step entails defining an appropriate estimand based on the predetermined study objective. The clinical study can effectively address specific questions and precisely define the treatment effect based on the insights gained from the first step with the defined estimand. The third step involves quantifying the well-defined treatment effect through primary and sensitivity analyses. In addition, researchers can incorporate supplementary analyses to provide a comprehensive understanding of the outcomes. Following this framework, researchers can coordinate the consistency between the study’s objective and implementation, foster effective communication among stakeholders, and enhance the overall quality and interpretation of the clinical study results.

The most crucial concept in E9(R1) is estimand. It addresses the clinical question by describing the treatment effect at the population rather than the individual level. When comparing the treatment effects among different groups, it is crucial to ensure that the estimated effect purely results from the treatment differences rather than confounding factors such as baseline differences between the treatment groups or variations in trial implementation.

The estimand consists of five attributes: population, treatment, variable or endpoint, ICEs and their handling strategies, and the population-level summary. Please refer to the E9(R1) guidance for detailed explanations of these attributes. E9(R1) consolidates these attributes into the concept of “estimand” rather than focusing solely on variables. It is important to note that these attributes are interconnected rather than isolated. The determination of the five attributes relies on the clinical question, particularly the ICEs that may occur during the trial. Therefore, when the trial objective may not be apparent at the outset of the clinical trial design, discussing the five attributes can help clarify the trial objective further.

### 2.2 Intercurrent events and handling strategies

In the estimand framework, ICE plays a crucial role. It encompasses events that may occur after treatment initiation that can affect clinical interpretations of the observed treatment effect or the existence of the measurements associated with the clinical question of interest. ICE is interconnected with other attributes of estimand. For example, the use of rescue medication is linked to treatment; treatment switching is associated with the population. Of note, ICEs cannot be removed by randomization; unlike protocol deviations, the occurrence of ICEs is inevitable and does not indicate flaws in trial design or implementation. However, the strategies for handling them should be carefully considered during the trial design stage. ICH E9(R1) introduces five commonly used strategies to address them, including treatment policy strategy, hypothetical strategies, composite variable strategies, while on treatment strategies, and principal stratum strategies. The selection of a suitable strategy in practical applications depends on the objective and specific circumstances of the trial.

Long-term clinical practice has established relatively comprehensive design rules in anti-cancer drug research. The associated guidelines provide us with ideas on how to handle ICEs. An example is present in the FDA guidance “Clinical Trial Endpoints for the Approval of Non-Small Cell Lung Cancer Drugs and Biologics,” which provides various censoring rules for PFS. According to this guidance, patients receiving new anti-cancer therapy before experiencing disease progression or death can be censored at the last tumor assessment for the PFS endpoints. In the estimand framework, it can be considered a hypothetical strategy. Conversely, during the follow-up period after the occurrence of an ICE, if patients experience disease progression or death, this is regarded as the occurrence of the endpoint, representing the treatment policy strategy. These guidelines offer valuable insights into different approaches for addressing ICEs and selecting appropriate treatment strategies in clinical trials for anti-cancer drugs.

The endpoints used to assess the long-term efficacy of anti-cancer therapies, such as OS, PFS, time-to-second objective disease progression, and disease-free survival, are typically time-to-event data, measuring the time from randomization or treatment initiation to the occurrence of the clinical event of interest. If ICEs occur before the observation of the endpoint, researchers have various approaches for handling them. For example, they can censor the data at the time of the unexpected event’s occurrence or treat it as a competing risk. Alternatively, the endpoint can be combined with the other events to construct a composite endpoint. It is essential to highlight that the events can be either ICEs or events that lead to missing data. Both types of events can be effectively handled through censoring. In the trial design stage, the investigators should differentiate between ICEs and events leading to missing data and select appropriate strategies for addressing them.

Within the E9(R1) framework, it is essential to proactively assess all potential ICEs during the trial design stage and establish appropriate strategies to address them. When dealing with time-to-event clinical endpoints, censoring is a commonly used method. For instance, patients who receive new anti-cancer therapy may be censored at their last efficacy assessment. However, the feasibility of the censoring strategy relies on the assumption that the probability of observing the clinical endpoint is the same for censored and uncensored patients, i.e., non-informative censoring. Nevertheless, these ICEs can indeed influence the probability of event occurrence. For instance, treatment switching may lead to informative censoring when assessing OS. In a randomized trial, a patient who responds poorly to his/her allocated treatment or experiences severe adverse events may switch to the other treatment due to ethical considerations. In this scenario, it is reasonable to anticipate an improved OS after treatment switching (Jin and Fang, 2023). Thus, it is necessary to determine whether censoring based on ICEs is non-informative or informative and employ suitable statistical methods for data analysis. Furthermore, the determination of handling strategies should be founded on the characteristics of specific ICEs and the applicability of censoring rules rather than blindly employing the censoring rule outlined in existing regulatory guidance since the provided rules may only cover some possible scenarios encountered in real-world practice. The results obtained using these rules may only partially address the clinical questions of interest. Therefore, it is necessary to carefully consider the importance of ICEs and censoring rules in the context of the specific clinical goals and adapt the strategies accordingly.

The choice of strategy for handling them should be based on a precise definition and comprehensive discussion regarding the trial objectives and the corresponding estimands. For example, if OS is the primary efficacy endpoint in a clinical study, the occurrence of receiving new anti-cancer therapies is a common ICE. If a patient is treatment-naïve, various subsequent treatment options are available after disease progression or intolerance or even participation in another clinical trial. In such cases, the receipt of new anti-cancer treatments can significantly influence the estimation of OS in the current clinical study. Therefore, employing a hypothetical strategy and censoring when receiving a new anti-cancer treatment is feasible. On the other hand, last-line patients have limited subsequent treatments with a substantial impact on efficacy after disease progression or intolerance. In this scenario, adopting a treatment policy strategy is reasonable. By carefully considering the characteristics of the patient population, the availability of treatment options, and the potential impact on estimating the treatment effect during the trial design stage, researchers can determine the appropriate strategies for addressing ICEs.

## 3 Choosing an appropriate endpoint

In late-phase clinical studies of anti-cancer therapies, conventional anti-cancer treatment clinical studies are commonly employed, including objective response-related and long-term benefits-related measurements (Anagnostou et al., 2017). Objective response endpoints are often considered surrogate endpoints for long-term benefits. Long-term benefit endpoints mainly include PFS and OS, with the former usually used when the prognosis of the tumor type is relatively long.

### 3.1 Short-term benefits V.S. long-term benefits

In the past 25 years, regulatory authorities have granted conditional approval to many anti-cancer drugs based on the promising outcome associated with ORR. However, subsequent studies are necessary to demonstrate long-term efficacy (Beaver et al., 2018). A promising objective response is the first step in demonstrating the clinical efficacy of an anti-cancer treatment. A fundamental assumption of using objective response as a surrogate endpoint of long-term efficacy is that the two are closely correlated, which has strong evidence in chemotherapy and targeted therapy (Burzykowski et al., 2008; Blumenthal et al., 2015; Topalian et al., 2023). Nevertheless, a similar relationship has not been conclusively established for immunotherapy and other emerging therapies (Roviello et al., 2017; Kaufman et al., 2018). The uncertain relationship between short-term and long-term endpoints is led by multiple reasons, such as the mechanism of action (Ritchie et al., 2018), late/delayed effects, and quality of life. Furthermore, the limited sample size (Paz-Ares et al., 2017) and population heterogeneity across different clinical studies may reverse the clinical trial results. On the other hand, the relationship between duration of response (DOR) and long-term endpoints, i.e., PFS and OS, is also unclear. Therefore, it is critical to verify the long-term benefits based on the positive ORR and long DOR for immunotherapy.

### 3.2 Novel long-term endpoints

Probably, time-to-event data do not adhere to the proportional hazards (PH) assumption, leading to inaccurate estimates of commonly used metrics, such as median OS and median PFS. Furthermore, anti-cancer therapies have the potential to provide extended survival benefits, necessitating longer follow-up periods compared to conventional treatments. The delayed effects of these novel therapies pose challenges in interpreting their benefits using conventional measures such as hazard ratio (HR), median OS, and median PFS. Additionally, due to the complex mechanism of action, specific therapies may primarily impact the later stages of cancer progression or metastasis, which are more relevant to OS, rather than the early stages of tumor progression (i.e., PFS) (Hess et al., 2019). Therefore, anti-cancer therapies may demonstrate a significant OS benefit while an insignificant PFS benefit, especially in a relatively short follow-up time. In a clinical study investigating the therapeutic effects of nivolumab *versus* docetaxel in advanced non–squamous non–small–cell lung cancer (Brahmer et al., 2015), the results highlight a significant advantage favoring nivolumab in terms of OS. Nivolumab exhibits a substantial extension in median OS by 3.2 months. In contrast, when examining PFS, the results do not support nivolumab. Patients receiving nivolumab experience a median PFS of 0.7 months shorter than those treated with docetaxel. Adjustments to sample size and statistical models are also necessary when the data severely violate the PH assumption. These factors collectively present challenges in evaluating the long-term efficacy of anti-cancer therapies.

One approach to address the abovementioned issues is to employ milestone survival analysis (Chen, 2015). It is vital to distinguish milestone survival analysis from the Landmark method (Anderson et al., 1983), as they are distinct techniques. Milestone survival analysis involves conducting cross-sectional analyses of survival data at predefined time points, allowing for qualitative or quantitative assessments. When utilizing milestone survival analysis, we should carefully select the appropriate time points, as this method may not capture the long-term benefits of the experimental treatment. Typically, milestone survival analysis is conducted as an interim analysis and includes multiple time points, such as 1-year survival rate and 2-year survival rate. Therefore, it is necessary to consider alpha spending in this strategy.

In addition, restricted mean survival time (RMST) has gained significant attention as an endpoint in anti-cancer therapies, mainly when the PH assumption is unmet (Royston and Parmar, 2013; A’Hern, 2016). RMST is the area under the survival curve up to a specific time. The critical issue in utilizing RMST is the selection of time, which should be carefully determined during the planning stage of a clinical study (Hasegawa et al., 2020). The choice of time point can be based on clinical expertise or the overall trial duration (Tian et al., 2020).

Net survival benefits is a statistical method that utilizes generalized pairwise comparison to assess treatment outcomes (Péron et al., 2019). It is the difference between the probability that patients in the experimental group survive longer than those in the control group and the opposite scenario in a randomized clinical trial (Péron et al., 2018). A positive net survival benefit indicates that the experimental group outperforms the control group, while a negative value suggests the superiority of the control group. The investigators can also evaluate the net survival benefit within a specific time frame. For instance, the 6-month net survival benefit measures the difference between the probability of a randomized patient in the experimental group surviving at least 6 months longer compared to the control group. It is important to note that the estimated net survival benefit can be influenced by the duration of follow-up, particularly when the time to event extends beyond the follow-up period.

### 3.3 Patient-reported outcomes

As the subjects of clinical trials, patients have the most direct experience of their disease state and treatment process, making them a valuable source of information during the drug development procedure. The concept of “patient-focused” has emerged as a crucial guiding principle in drug research, emphasizing patient needs and clinical value as the ultimate goal. Regulatory agencies are proactively promoting strategies to design and implement “patient-focused” clinical trials while incorporating patient’s perceptions into the benefit-risk assessment of drugs. In line with this, the NMPA issued guidance for industry (NMPA, 2022a; NMPA, 2022b; NMPA, 2022c; NMPA, 2022d; NMPA, 2022e; NMPA, 2023), including “General considerations for involving patients in drug development” and “Technical guidance for patient-focused clinical trial design.” Meanwhile, the FDA has issued eight guidelines on this topic (FDA, 2016; FDA, 2018; FDA, 2021; FDA, 2022a; FDA, 2022b; FDA, 2022c; FDA, 2023), including “Patient-reported outcome measures: Use in medical product development to support labeling claims: Guidance for industry,” “Core patient-reported outcomes in cancer clinical trials: Draft guidance for industry.” These guidelines reflect regulatory considerations on integrating patient’s perceptions into evaluating drug efficacy and safety. ICH has also developed patient-focused initiatives. In 2020, ICH conducted a public consultation on its “Reflection Paper on Patient-Focused Drug Development (PFDD)," which explores including patient’s perceptions throughout the drug development process.

In clinical trials, it is essential to incorporate patient experience data and prioritize patient’s perceptions into design considerations while adhering to conventional clinical trial design principles. By doing so, the clinical trial can better reflect patient clinical benefits. Firstly, patient needs are foremost throughout the drug development process. Researchers are encouraged to engage with patients and continuously seek their input throughout the lifecycle to identify unmet clinical needs. Furthermore, a patient-focused clinical trial should comprehensively assess the clinical benefits for patients in terms of their physical and mental wellbeing, functionality, and quality of life. This involves considering the research objectives, target population, control selection, and safety and efficacy evaluation methods from the patient’s perceptions. Additionally, trial designs should adopt a format acceptable to the patients to reduce participant dropout rates, improve subject representativeness, and enhance compliance. While maintaining scientific rigor and integrity, the trial design should strive to provide convenience and minimize the burden on patients. This can enhance the overall patient experience in the trial.

In patient-focused clinical research, PROs are essential tools. PROs refer to the status of a patient’s health condition that comes directly from the patient, without interpretation by a clinician or anyone else (FDA, 2009; NMPA, 2022b). PRO assessments should come directly from the patients themselves. In cases where patients cannot self-report, the investigators may consider using proxy reports. However, in such situations, it is essential to evaluate the bias that proxy reports may introduce carefully. The extent of bias introduced by proxy reports in a specific PRO measurement hinges on various factors, including the indication, the patient population, the measurements, and other clinical characteristics. Therefore, the acceptability of proxy bias should be evaluated on a case-by-case basis, including assessing the concordance between patient-reported and proxy-reported outcomes through a clinical study that compares both types of reports for the same individuals (Sneeuw et al., 1998; Duncan et al., 2002; Roydhouse et al., 2018). If the difference between these two sets of outcomes is negligible, the bias introduced by proxy reports may be deemed acceptable. However, if substantial disparities exist, the clinical study should implement measures to mitigate this bias through statistical methodologies, consider excluding the outcomes provided by proxies, or explore alternative objective measurements. When measuring PROs, scales are usually helpful in evaluating various aspects, such as pain intensity and quality of life. The first choice is to employ well-established and validated scales whenever possible. However, if suitable scales are unavailable, developing new scales tailored to the research objectives may be necessary. This includes considering the scale’s relevance for evaluating treatment efficacy, its interpretability in terms of clinical value, and its ability to guide treatment decision-making. Before implementation, the newly developed scale should undergo rigorous validation, including pre-investigations among the target population, to ensure its reliability and validity. Reliability refers to the consistency of scale results under similar conditions, indicating the scale’s robustness. Conversely, validity assesses the extent to which the scale accurately measures the intended attribute or concept, demonstrating its effectiveness in capturing the construct of interest. To ensure accuracy, it is essential to thoroughly plan and develop PRO measurements during the design phase to guarantee their reliability, validity, and capacity to detect meaningful changes. From an operational perspective, specific vital characteristics, including the concepts, number of items, as well as the conceptual framework of the instrument, demand careful attention. For a more comprehensive understanding of assessing the appropriateness of a PRO measurement, we recommend that readers refer to the guidance provided by regulatory agencies. It is essential to acknowledge that PRO measurements are inherently subjective, as they rely on patients’ self-reported experiences and perceptions. Therefore, we do not anticipate complete objectivity in patients’ responses. However, a well-designed PRO measurement should possess the necessary validity to generate accurate estimates of the targeted concept of interest.

In anti-cancer research, the benefit-risk evaluation for anti-cancer treatments traditionally focuses on survival benefits, tumor remission, prevention of tumor progression, and physician-evaluated adverse events. However, with the advancements in drug development, cancer patients are now experiencing prolonged survival, and some patients can even achieve lifespans comparable to those of healthy individuals. As a result, patients’ experiences and quality of life during the treatment journey gain increasing importance in clinical research on anti-cancer drugs, aligning with “patient-focused” care. In clinical research for anti-cancer treatments, a range of PRO measurements can be employed to capture the patient’s perceptions of their disease and treatment experience. Disease symptoms, symptomatic adverse events, and physical function are among the core PROs contributing to a patient’s health-related quality of life. They can be sensitive to the disease progress and treatment effect. Patient-reported disease-related symptoms provide valuable insights, especially when there are variations in the type and frequency of symptoms among patients (Kluetz et al., 2016). Direct reports from patients regarding symptomatic adverse events yield additional safety data beyond that in traditional clinical studies. However, it is crucial to carefully select the assessment items based on the treatment’s mechanism of action, early clinical data, and patient and healthcare provider input. Additionally, capturing a summary measure of the side effects can be valuable in assessing the overall tolerability of a treatment. Furthermore, evaluating physical function, which refers to a patient’s ability to perform activities, and role function, which relates to their ability to work and carry out daily tasks, are also essential aspects of PRO evaluation (FDA, 2021).

Another important consideration when utilizing PRO measures for anti-cancer treatments is the frequency of assessment. Since patients typically receive anti-cancer drugs in cycles, PRO measures should align with these treatment cycles, with varying emphasis on different measures. For instance, assessing symptomatic adverse events may be more frequent during intensive drug use. On the other hand, physical function and role function measures can serve as representative indicators of patients’ functional preservation or improvement. Therefore, conducting more frequent assessments of these measures is advisable when the drug is highly probable to demonstrate efficacy. By considering the treatment cycles and the specific outcomes of interest, researchers can optimize the assessment frequency of PRO measures, thereby capturing the relevant patient experiences and treatment outcomes more comprehensively. To prevent data bias, the researchers should elaborate on the methods for addressing potential issues, such as missing data, before receiving patients’ reports. Establishing criteria for valid responses and determining appropriate methods for handling missing values within the trial protocol are essential steps. By addressing these considerations, the integrity and reliability of the PRO data can be guaranteed, ensuring a more accurate representation of patients’ experiences and outcomes in anti-cancer clinical trials.

Including PRO results in drug labeling requires high-quality data and rigorous study design and implementation. Several considerations are necessary to ensure the validity and meaningful interpretation of PRO results. Firstly, when comparing PRO results between treatments, conclusions of “no meaningful difference” should be based on non-inferiority or equivalence testing rather than superiority testing. Secondly, since PRO assessments often encompass multiple measurements, it is crucial to address the issue of multiplicity and control type I error. Additionally, exploratory PRO results that were not pre-specified in the statistical analysis plan generally cannot be included in the drug labeling, even if they demonstrate promising outcomes. The regulatory agencies will carefully evaluate the appropriateness of including such results in the labeling.

## 4 Long-term efficacy evaluation

### 4.1 Non-proportional hazard (NPH) assumption

PFS and OS typically serve as primary efficacy endpoints in clinical studies when assessing long-term efficacy. Commonly employed statistical methods to evaluate these endpoints include the log-rank test and the Cox PH model. Additionally, researchers employ the Kaplan-Meier (KM) method to construct the survival curves and estimate the median survival time. It is important to note that these methods rely on the PH assumption, which implies that the HR remains constant over time.

However, in the context of anti-cancer therapies, time-to-event data usually do not satisfy the PH assumption. Several reasons can lead to this violation: 1) Delayed effect: There is often a prolonged delay before the survival curves begin to separate. For immunotherapy, this delay results from the complex mechanisms of immunotherapy and immune resistance. 2) Gradual decrease in treatment effect: The survival curves tend to converge over time, indicating a diminishing treatment effect. The decrease of treatment effect in the later stage is due to the natural development of indolent tumors in patients who are not sensitive to anti-cancer therapies (Maio et al., 2015). 3) Crossing hazards: The survival curves intersect or cross at a specific time. This crossing can occur due to delayed effect or hyperprogression in patients (Borcoman et al., 2019). The crossing of survival curves suggests that there may be variables or factors that significantly impact the treatment outcomes. In other words, different subgroups of patients may experience distinct treatment effects (Mok et al., 2009).

In practice, there will always be some degree of violation. The log-rank test will still be valid in case of violation, although it may not be optimal. HR estimate of the Cox regression model would be a weighted estimate of hazard ratio over time, which is meaningful to a certain degree, especially when the violation is minor. Besides, since these methods have been used to analyze historical trials, applying them to new trials makes the comparison fair. We should continually improve the analysis methods. However, when the data suggests a significant violation of the PH assumption, analyzing time-to-event data using statistical methods based on such an assumption can lead to several issues, including reduced statistical reliability (Chen, 2013), unreliable interim analysis results (Chen, 2015; Korn and Freidlin, 2018), and challenges in interpreting final results (Pak et al., 2017; Liang et al., 2018). Consequently, improving existing statistical methods or developing new ones to address the challenges is necessary. To suit NPH assumptions, the development of efficacy analysis focuses on hypothesis testing methods and efficacy estimation methods tailored to different NPH scenarios. When evaluating new statistical methods, there are various perspectives to consider, with two important ones being: 1) Impact on type I and type II errors: It is essential to assess whether the new method increases the probability of committing type I or type II errors. Controlling error rates at acceptable levels is crucial for the validity and reliability of statistical inference. 2) Applicability across diverse scenarios: The new statistical method should exhibit stable performance across different situations. It should be adaptable and effective in handling various types of NPH, ensuring its utility and reliability in a wide range of research settings since the true nature of the data cannot be predicted in advance. By addressing these considerations, researchers can strive to develop robust and versatile statistical methods that overcome the challenges posed by NPH in analyzing time-to-event data.

### 4.2 Hypothesis testing in the presence of NPH

Several hypothesis testing methods are available for NPH models, including the weighted log-rank and the RMST-based tests. Furthermore, additional methods are specifically designed to evaluate the efficacy of anti-cancer therapies (Ye and Yu, 2018; Ding and Wu, 2020).

The weighted log-rank test extends the standard log-rank test by incorporating a time-varying weighting function. By using various function forms and parameters, the weighted log-rank test assigns different weights to different parts of the data, allowing for the consideration of varying HRs over different time intervals (Zucker and Lakatos, 1990; Lin and León, 2017). Specifically, we can assign higher weights to the parts of data that we focus on. In delayed effects, assigning more weight to later data can increase the trial’s statistical power (Fine, 2007; Thomas and Fleming, 2011). However, the weighted log-rank test has certain limitations. It is challenging to evaluate the suitability of the selected weighting function before observing the actual data, whereas determining the weighting function after obtaining the outcomes would violate the principles of clinical trial design. Such ad-hoc analysis is unlikely to be accepted by regulatory agencies. Additionally, interpreting the results generated by the weighted log-rank test is more complex. It can be challenging to translate significant test results obtained by weighting data from different periods into meaningful clinical conclusions, especially when conventional log-rank tests fail to yield significant results. It is also essential to contemplate the potential issue that various weighted log-rank tests could produce divergent results (Royston and Parmar MK, 2020). Furthermore, we should also acknowledge the risk that weighted log-rank tests may reduce statistical power if the PH assumption holds (Karrison, 2016a).

The RMST-based test compares the area under the KM curves, and the difference in RMST between groups can be tested using a *t*-test assuming normal distribution (Hasegawa et al., 2020). Therefore, the test’s significance relies heavily on the selection time point τ. Currently, there are various methods for choosing τ in RMST analysis, some of which aim to maximize the difference in RMST between groups (Andersen and Pohar Perme, 2009; Guyot et al., 2012). However, this approach also introduces confusion regarding whether the RMST based on the chosen time point represents clinically meaningful treatment efficacy.

In the design of clinical trials, pre-specifying statistical methods are crucial to increase the reliability of trial results. However, when evaluating anti-cancer therapies that do not meet the PH assumption, accurately pre-specifying the hypothesis testing method becomes challenging due to the unpredictable nature of the time-to-event data. To address this issue, some researchers have attempted to pre-specify multiple tests by using a versatile testing approach at the design stage. For example, the $G^{\rho ,\gamma }$ family of weighted log-rank tests (Lee, 1996) assigns different weights to survival data in different stages under different values of ρ and γ. The test statistics $G^{0,0}$ , $G^{1,0}$ , and $G^{0,1}$ can be combined (Karrison, 2016b). Simulation results have shown that the statistic $Z=\max\left(\left|Z^{0,0}\right|,\left|Z^{1,0}\right|,\left|Z^{0,1}\right|\right)$ can better control the type I error compared to other joint testing methods (Chi and Tsai, 2001; Royston and Parmar, 2016). Furthermore, versatile testing methods offer advantages in retaining statistical power. The essence of joint testing is to test different scenarios under the NPH assumption of time-to-event data and select the scenario most likely to reach statistical significance. This approach helps reduce the false negative rate and identify the treatments with the largest potential to have therapeutic effects.

### 4.3 Efficacy evaluation in the presence of NPH

Estimating efficacy plays a crucial role in conducting risk-benefit assessments for medical interventions. In cases where the PH assumption can no longer hold, there are two strategies to improve efficacy estimation: modifying commonly used methods and establishing new ones. By employing these strategies, researchers can obtain a more accurate assessment of the benefits associated with the experimental treatment.

The commonly used efficacy endpoint is the HR, estimated by the Cox PH model. To mitigate the risk of NPH, alternative modifications of the Cox model can be considered, such as the stratified Cox model and the time-dependent covariate Cox model (Kleinbaum and Klein, 1996), to generate more accurate and reasonable results. Various methods can be employed to examine whether a covariate violates the PH assumption. Firstly, experts’ opinions can provide preliminary knowledge about which covariates are likely to violate the PH assumption. Statistically, we can use graphical approaches (e.g., log-log plots, observed *versus* expected plots) and hypothesis testing approaches (e.g., good-of-fit testing) to check the PH assumption (Kleinbaum and Klein, 1996). A stratified Cox model can be used when some covariates meet the PH assumption while others do not. Specifically, we put the patients with the same covariates that do not satisfy the PH assumption into one stratum. By doing so, the PH assumption is satisfied within each stratum, so the Cox PH model can work in a single stratum. The stratified Cox model allows for different baseline hazards for each stratum, so the between-group differences in a trial consist of differences at baseline and in covariates incorporated in the model. Under the no-interaction assumption, the HR of each covariate is consistent at different strata. That is, the covariates and their coefficients included in the models of different strata are the same. In practice, if there is still uncertainty about the interaction between the covariates incorporated in the model and that used for stratification, rigorous sensitivity analyses may be helpful to assess the feasibility of the no-interaction. When using a stratified Cox model, a key consideration is that a sufficient number of events within each stratum is needed to ensure the robustness and reliability of the analysis. Besides, obtaining a conclusive result for the whole population through the stratified Cox model is difficult, especially when the no-interaction assumption is not satisfied. This is a crucial problem in clinical research.

The covariates are assumed to be time-independent in the standard and the stratified Cox models. However, we can also extend the Cox model by incorporating time-dependent covariates, allowing for a more comprehensive analysis in complex cases. The extended Cox model for time-dependent variables produces a time-varying HR by introducing time-dependent covariates. It allows for a more accurate representation of the dynamic nature of the covariates and their impact on survival outcomes. The extended Cox model for time-dependent variables offers greater flexibility in analyzing survival data. Still, it is also more complex compared to standard and stratified Cox models. Despite its advantages, the extended Cox model presents several challenges. One is the selection and precise definition of time-dependent variables. Careful consideration and expertise are required to determine the appropriate time-dependent variables in a clinical study. Another challenge is interpreting the model when endpoint changes result in covariate changes. This situation can make understanding the relationship between the variables and their impact on the outcome more challenging. Additionally, the extended Cox model faces limitations in predicting the future status of patients. Since the model relies on the available covariate information, it cannot predict the status of a patient at future time points when the covariates are unknown (Fisher and Lin, 1999).

It is essential to highlight that when employing variations of the Cox model, their application should be grounded in clinical judgments rather than pursued solely to mitigate the risk of NPH. For instance, consider stratified analysis founded on the premise that the levels of particular covariates impact the experimental treatment’s effectiveness. We should identify the causes of the PH assumption breach and take suitable measures. On the other hand, every statistical analysis method carries its own set of advantages and disadvantages. While the modified Cox models can be employed to tackle broader issues, it is also important to acknowledge their inherent limitations. As a result, we should utilize these methodologies with careful consideration.

As discussed in Section 3, several novel efficacy endpoints are available for estimating long-term efficacy, among which RMST is one of the most widely discussed (Blumenthal et al., 2015; Topalian et al., 2023). In practice, it supports descriptive statistics and interim analyses of cancer therapies. For example, a randomized controlled trial investigating trastuzumab as adjuvant therapy for HER2-positive elderly patients who did not receive chemotherapy employed RMST (Sawaki et al., 2018). The between-group difference in RMST can be supplied as a complementary efficacy estimate (Eng et al., 2015). RMST is similar to the median survival time, but the clinical significance of its results depends on the specification of the time point τ . However, the experience of applying the RMST is currently limited due to the absence of a standardized method for determining the optimal time point τ .

Other efficacy measures, such as net survival benefit, can be utilized as complementary indicators to primary efficacy estimates. The clinical interpretation of net survival benefit is straightforward, as it represents the probability of experiencing a longer survival time after receiving treatment in the experimental group compared to the control group.

Once again, it is essential to recognize that encountering a violation of the PH assumption is not uncommon in practical applications. Taking measures to counter the risk of NPH could bring forth new challenges. In implementing pioneering methodologies designed to tackle NPH, it is crucial to balance the potential risks and benefits carefully. Therefore, an acceptable approach could entail adopting conventional analytical methods as the primary analysis, complemented by exploring ancillary analyses employing alternative methodologies.

### 4.4 Treatment switching

Treatment switching refers to the process in a clinical trial where patients discontinue their assigned experimental or control treatment and switch to an alternative therapy according to the protocol. This alternative treatment can be the other treatment incorporated within the trial, such as changing from the experimental group to the active control group or *vice versa*. Alternatively, it can involve other treatments used in clinical practice, such as new anti-cancer therapies, later-line treatments, or best supportive care. More complicated scenarios may arise in actual clinical studies. For example, patients may undergo multiple switches, such as transitioning from the control to the experimental group and then to a later-line treatment. However, these complex scenarios are beyond the scope of this paper. Currently, limited specific guidance is available regarding switching treatments in clinical trials. The European Medicines Agency has published a question-and-answer document that provides advice on switching methods.

In the study of anti-cancer drugs, the impact of treatment switching varies depending on the endpoint. If treatment switching occurs after the occurrence of the event of interest, it typically does not affect the efficacy evaluation. For example, in a case where the primary efficacy endpoint is PFS, and treatment switching is only allowed after the occurrence of disease progression, it does not impact the evaluation of PFS. However, if treatment switching happens before the occurrence of the event of interest, it can have an impact on efficacy evaluation. For instance, if the primary efficacy endpoint is OS, patients may undergo treatment switching for various reasons before death, and the effect of switching treatment on efficacy evaluation can be substantial. Therefore, to maintain the integrity of the trial, it is generally not recommended to allow treatment switching before observing the endpoint. Otherwise, treatment switching can significantly influence the assessment of treatment effect, and analyzing its impact can be challenging. However, providing more effective treatments to more participants in clinical research is ethically more appropriate. Therefore, when treatment switching occurs and impacts the efficacy evaluation, appropriate handling strategies and statistical methods should be employed to assess this impact.

There are two main approaches to addressing the treatment switching. The first approach is the non-counterfactual method, which relies on objective and observable clinical factors (such as treatments received and crossover) to estimate the overall survival benefit of the experimental treatment compared to the control treatment. Non-counterfactual methods are relatively simple, and their conclusions are easily understood, making them commonly used in current clinical trial methodologies. Standard methods include intention-to-treat (ITT), per-protocol method, censoring method, as-treated method, crossover design (Law and Kaldor, 1996), and inverse probability censoring weighting (Hernán et al., 2000).

The second approach is the counterfactual method, which is based on hypothetical conditions (such as assuming no crossover occurred) to evaluate the treatment effect difference of the experimental drug on the target population. However, this hypothetical condition is usually unobservable in clinical studies, and the validity of its results often relies on whether the causal estimand can meet the basic assumptions of consistency, positivity, and exchangeability. Standard counterfactual methods used to adjust for crossover include the two-stage estimation method (Latimer et al., 2016), rank-preserving structural failure time (RPSFT) model, and iterative parameter estimation method (IPE). The specific concepts of these methods are not described in this paper. The methods above are not commonly used as primary analysis methods. However, they often serve as sensitivity analyses to evaluate the robustness of efficacy results or when the event of interest is a secondary efficacy endpoint.

The approach to address treatment switching should align with the research objective, precisely the scientific question of interest. The feasibility of implementing crossover rules depends on multiple factors, such as the trial design, clinical endpoint, and indication. In the clinical trial design, careful consideration and comprehensive demonstration of the necessity and feasibility of allowing treatment switching are crucial. A complete overview of strategies and statistical analysis methods for handling treatment switching in different clinical scenarios has been provided (Manitz et al., 2022). For instance, if treatment switching reflects real-world clinical practice, the treatment policy strategy is a reasonable choice, and the conventional Cox models or KM methods are appropriate for the primary analysis. On the other hand, if the main reason for treatment switching is disease progression, it is necessary to adjust the observed treatment effect using hypothetical strategies. Specifically, counterfactual survival times are applicable for estimating HRs or modified KM through a two-stage method. The IPCW and RPSFT methods may also be suitable. The treatment policy strategy is still suitable for treatment switching caused by other reasons. In summary, selecting an appropriate evaluation method for treatment switching should consider the specific research objectives, trial design considerations, and statistical approaches that best address the clinical questions of interest.

In addition, it is crucial to evaluate the impact of treatment switching on trial integrity and develop appropriate methods to safeguard trial integrity if necessary. We recommend that the sponsors communicate with regulatory agencies regarding the protocol details before conducting the pivotal clinical studies.

## 5 Discussion

As research in anti-cancer treatment advances, the evaluation of the benefits of tumor drugs develops accordingly. Prolonging patients’ survival time remains the ultimate goal, and it is crucial in assessing treatment benefits. To address unmet clinical needs, regulatory agencies may grant marketing approvals to specific drugs based on promising short-term outcomes, such as tumor remission, to expedite the drug approval process and enable patients to access new treatments more quickly. However, positive responses in surrogate endpoints may not indicate actual benefit. As more anti-cancer drugs are available, patients now have various treatment options. Therefore, regulatory agencies will evaluate the benefit-risk of drugs based on solid evidence, focusing on significant long-term benefits. On the other hand, the correlation between short-term and long-term efficacy varies among different types of anti-cancer drugs. For cytotoxic drugs, significant outcomes of short-term efficacy endpoints often provide substantial evidence indicating long-term benefits. However, such a positive correlation only sometimes holds in the case of immunotherapy. Despite achieving positive results in ORR and DOR, it is essential to verify the long-term benefits through additional clinical trials. Besides, in cell therapy, patients may experience rapid response, but the investigators should be mindful of potential relapses. Evaluating long-term benefits becomes even more critical, given anti-cancer therapies’ intricacy and diverse characteristics.

In evaluating long-term efficacy, a severe violation of the PH assumption will render the commonly used statistical methods for efficacy evaluation inadequate. Several efficacy endpoints and statistical methods have been developed to address this issue, including milestone survival analysis, RMST, net survival benefit, joint tests, time-varying covariate Cox models, and accelerated failure time (AFT) models. Each method has advantages and limitations but has yet to be widely accepted as the primary statistical method for market approval applications (Buyse et al., 2020). However, it is worth noting that each efficacy endpoint provides valuable insights into the clinical benefits of a treatment from a specific perspective. Therefore, we recommend considering these novel endpoints as secondary evidence when evaluating the clinical benefits of a treatment. For instance, in a clinical trial (NCT02506153) investigating the treatment of high-risk stage III-IV melanoma patients who underwent surgical resection, high-dose recombinant interferon alfa-2b, ipilimumab, or pembrolizumab, RMST was employed to assess the primary efficacy endpoints, OS and recurrence-free survival (RFS). Another example is the Lung-Map study (NCT05096663) comparing the efficacy of combined immunotherapy and conventional treatment in patients with advanced non-small cell lung cancer. In this study, both conventional log-rank and weighted log-rank tests were employed to evaluate the primary efficacy endpoint, OS.

For researchers, violating the PH assumption when using conventional statistical methods can lead to an unexpected decrease in statistical power. On the other hand, using methods specifically designed for NPH can introduce challenges in clinical interpretation. Therefore, it is crucial to thoroughly evaluate different statistical methods and select appropriate ones during the planning stage of a clinical study. Furthermore, studies have suggested several strategies to address the issue of power reduction caused by violations of the PH assumption. These strategies include increasing the sample size by 10% (Hoering et al., 2017), conducting additional follow-up (Pak et al., 2017), or implementing adjusted interim futility analysis (Korn and Freidlin, 2018). By adopting these approaches, the impact of NPH can be mitigated, and the study’s statistical power can be improved.

With increasing available treatment options, assessing long-term efficacy becomes even more critical for patients to make informed decisions regarding the optimal treatment therapy. To approach this, the framework of E9(R1) offers a valuable tool to describe the clinical question precisely. This involves comprehensively assessing potential ICEs and their corresponding handling strategies to describe long-term efficacy evaluations in specific real-world scenarios. These scenarios include situations where patients switch to the standard of care or receive the best supportive care. On the other hand, patients’ treatment switching can also impact long-term efficacy evaluation, leading to a potential loss of statistical power and an inaccurate estimation of treatment effects.

As medical advancements continue to unfold, we can expect the emergence of innovative statistical methods to address the current challenges in evaluating long-term efficacy and the discovery of more efficient therapies for cancer patients. We need to learn from past experiences and proactively promote the development of anti-cancer treatments.
